# A Molecular Marker to Identify *Spodoptera frugiperda* (JE Smith) DNA in Predators’ Gut Content

**DOI:** 10.3390/insects13070635

**Published:** 2022-07-15

**Authors:** Daniela Hipolito Maggio, Victória Zannuzzi Rossetti, Larissa Muniz Amaral Santos, Felipe Levorato Carmezini, Alberto Soares Corrêa

**Affiliations:** Luiz de Queiroz College of Agriculture (ESALQ), University of São Paulo (USP), Av. Pádua Dias, 11, Piracicaba 13418-900, SP, Brazil; dani.maggio@usp.br (D.H.M.); victoria.rossetti@usp.br (V.Z.R.); lmuniz@usp.br (L.M.A.S.); felipe.carmezini@gmail.com (F.L.C.)

**Keywords:** earwigs, ladybugs, half-life detectability, biological control, predator–prey interaction

## Abstract

**Simple Summary:**

This work aimed to build a molecular marker to detect *Spodoptera frugiperda* DNA in predators’ gut content. The molecular marker developed is highly specific, and it was able to detect *S. frugiperda* DNA in the gut content of ladybug and earwig predators in field conditions. Our results confirm that generalist predators feed on *S. frugiperda* in maize fields, and they must be considered in IPM programs for *S. frugiperda* suppression.

**Abstract:**

*Spodoptera frugiperda* is a serious pest of maize and other crops worldwide. The integration of control tactics is recommended for *S. frugiperda* suppression because reports of insecticide and Btplant-resistance are frequent. Biological control agents would be an alternative to improve *S. frugiperda* control in agricultural areas. We constructed a species-specific molecular marker to detect *S. frugiperda* DNA in predators’ gut content and estimated the predation rates of ladybugs and earwigs on *S. frugiperda* in maize crops. Predators were sampled in Pirassununga, São Paulo state, Brazil, in 2020 and 2021. Using the species-specific molecular marker in laboratory conditions, we estimated the half-life time to detect *S. frugiperda* DNA in the gut contents of *Hippodamia convergens* as 6.16 h and *Doru luteipes* as 25.72 h. The weekly predation rate of *S. frugiperda* by predators in maize crop varied from 0 to 42.1% by ladybugs and from 0 to 9.2% by *D. luteipes*. Predation events on *S. frugiperda* by predators were more frequent during the maize reproductive stage. Our results confirmed that predators might contribute to *S. frugiperda* suppression in maize fields. However, further studies of prey–predator interactions and agricultural landscapes are essential for a better understanding of predator dynamics in crops.

## 1. Introduction

*Spodoptera frugiperda* (JE Smith) (Lepidoptera: Noctuidae), popularly known as the fall armyworm, is native to the Americas. In 2016, *S. frugiperda* was detected in Africa and reached the status of a cosmopolitan species after invading and dispersing in regions of Europe, Africa, Asia, and Oceania [1,2,3]. Its polyphagous and voracious feeding behavior, high reproductive rate, and long-distance dispersal capacity make *S. frugiperda* a serious agricultural pest [4,5,6]. In Brazil, *S. frugiperda* has been an economically important pest of maize and cotton crops. However, in recent years, this species has increased in abundance in soybean crops, making its management more challenging [7].

Different tactics are used for *S. frugiperda* control, including insecticides, transgenic plants, and natural enemies. Insecticides are the most common management strategy for *S. frugiperda*. However, their effectiveness is limited because *S. frugiperda* larvae feed inside the maize whorl where insecticides may not penetrate [8]. Furthermore, in recent years insecticide-resistant populations of *S. frugiperda* have often been reported [9,10,11,12]. Transgenic *Bacillus thuringiensis* (Bt) plants are another worldwide control strategy for *S. frugiperda* management. However, as with insecticides, *S. frugiperda* populations resistant to Bt crops have been reported in Puerto Rico, Brazil, Argentina, and the USA [6,13,14,15].

Biological control agents may also be effective for *S. frugiperda* population suppression. Parasitoids and predators, such as *Telenomus remus* (Hymenoptera: Scelionidae), *Trichogramma* species (Hymenoptera: Trichogrammatidae), Tachinidae (Diptera), *Podisus nigrispinus* (Hemiptera: Pentatomidae), *Doru luteipes* (Dermaptera: Forficulidae), and *Harmonia axyridis* (Coleoptera: Coccinelidae), are described as promising biological control agents of *S. frugiperda* [16,17,18,19,20]. In maize areas, the most common predators are earwigs, ladybugs, ground beetles, and lacewings, which feed on different important agriculture pests [17,21,22,23].

Although predators are frequent in agricultural areas, the real contribution of these natural enemies to *S. frugiperda* suppression is still difficult to estimate in the field. One approach used to identify and estimate the predation rate of predators on a group of prey is an analysis of predator gut contents [24,25,26]. In the past, these analyses were carried out by identifying prey body parts found in the predator’s gut, although it was difficult to identify the prey to species level [24]. Therefore, molecular tools have been widely used in the analysis of predator gut contents because they allow rapid and precise identification based on the design of species-specific primers, multiplex PCR markers, and, more recently, metabarcoding [24,25,27,28].

Based on the worldwide economic importance and the reports of control failures of *S. frugiperda*, predators may be important components in the development of IPM plans to suppress this pest. We designed a specific molecular marker to detect the predation rate of generalist predators on *S. frugiperda* in maize crops. Specifically, earwigs and ladybugs are two groups of predators that are frequently cited as natural enemies of *S. frugiperda* [16,18], but they are inadequately studied in the field. Our specific aims were: (1) design species-specific primers to detect *S. frugiperda* DNA in the gut contents of generalist predators; (2) estimate the detectability half-life in *S. frugiperda* DNA in the gut contents of an earwig, *D. luteipes*, and a ladybug, *Hippodamia convergens* (Coleoptera: Coccinelidae)—both common generalist predators found in maize crops—and to confirm the utility of this molecular marker; and (3) estimate the predation rate of *S. frugiperda* for predators collected weekly in maize crops.

## 2. Materials and Methods

### 2.1. Field Detection of S. frugiperda and Predator Collection

Insect monitoring and collections were carried out on the maize farm in Pirassununga. The collections were made in two consecutive fields: in the winter, crop from April to July 2020, a total of 14 weeks of sampling; and in the summer, crop from December 2020 to February 2021, 8 weeks of sampling. In the winter crop, the maize plants developed more slowly due to the low rainfall (46 mm) and temperature (19 °C); consequently, the sampling period was longer than in the summer crop (mean rainfall 217 mm and mean temperature 25 °C). Sampling started in the maize vegetative stage V2 and continued until the harvest. The vegetative stage lasted until week 7 in winter and week 4 in summer. Both crops were cultivated under conventional practices but without insecticide applications for insect control.

A delta trap baited with the sex pheromone Bio Spodoptera (Bio Controle, Indaiatuba, Brazil) was installed in the middle of the area to detect the presence of *S. frugiperda* during the experiment. The sticky liner was replaced each week and the sex pheromone replaced every 3 weeks or less if the pheromone was depleted. In the laboratory, the moths on the sticky liner were photographed and identified using morphological traits such as body and wing colors.

Earwig and ladybug individuals were manually collected randomly along the plant each week from the maize vegetative stage V2 until the harvest. The maize crop area was divided into three sub-areas of 20 m^2^ each, and predators were collected actively to preserve their gut contents by immediately freezing the specimens. Each predator collected was immediately placed in a 1.5 mL tube and stored in a plastic bag on ice until arriving at the laboratory. At the laboratory, the predators were stored in 99.9% ethanol at –20 °C and taxonomically identified.

To estimate the predation rate by predators on *S. frugiperda*, the predators’ DNA was extracted, and the specific marker to detect the presence of *S. frugiperda* DNA in the predators’ gut contents was applied exactly according to the above method

### 2.2. Molecular Analysis of Gut Contents

#### 2.2.1. DNA Extraction

All insects used in this study were cleaned by submersion in 2% sodium hypochlorite for 2 s, next in 70% ethanol for 2 s, and then in autoclaved distilled water for 5 s and allowed to dry on clean tissue paper. These procedures were carried out to cleanse any external DNA from the insect body.

DNA was extracted using the entire body except for the wings, antenna, and legs, with the CTAB protocol adapted from Corrêa et al. [29]. Each individual was submerged in 500 µL of CTAB buffer, 10 µL of proteinase K 20 mg mL–1 (Invitrogen, Waltham, MA, USA), and 2 µL of β-mercaptoethanol. The samples were incubated at 65 °C for 2 h. Then, 3 µL of PureLinkTM RNase A 20 mg mL–1 (Invitrogen) was added to each sample, which was incubated at 37 °C for 2 h and then at 65 °C for 30 min. The samples were centrifuged at 14,000 rpm for 5 min and the liquid phase was transferred to a new tube, mixed with 500 µL of chloroform and isoamyl alcohol (24:1), and centrifuged at 14,000 rpm for 20 min. The supernatant was transferred to a new tube, and the isoamyl alcohol step was repeated. Then, the supernatant was transferred and mixed with 100% isopropanol and incubated at –20 °C overnight. The samples were centrifuged at 14,000 rpm at 4 °C for 30 min and the supernatant was discarded. The DNA pellet was cleaned with 400 µL of cold 70% ethanol. The cleaning step was repeated with cold 100% ethanol and then the samples were allowed to air-dry. The dried DNA pellet was resuspended in 30 µL of autoclaved distilled water.

#### 2.2.2. Primer Design and Optimization

**Figure 1:**
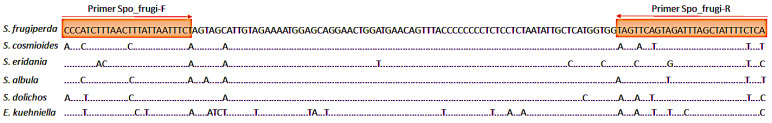
Part of COI gene sequences from *Spodoptera frugiperda*, *Spodoptera cosmioides*, *Spodoptera eridania*, *Spodoptera albula*, *Spodoptera dolichos*, and *Ephestia kuehniella*, aligned to design the pair of primers specific for *S. frugiperda*. Orange rectangles indicate the pair of forward (Spo_frugi-F) and reverse (Primer Spo_frugi-R) primers. Dots indicate pairs of bases similar to *S. frugiperda* COI sequences; letters indicate the respective different nucleotide bases in each species.

The *S. frugiperda* species-specific primers were designed using sequences of the *cytochrome c oxidase subunit I* (COI) gene available in GenBank (access numbers: KF624877.1, GU439148.1, JQ559528.1, JF854747.1, HQ964527.1, and MH753323.1) from different regions of Brazil and the world, Spo_frugi-F: CCCATCTTTAACTTTATTAATTTCT, and Spo_frugi-R: TGAGAAAATAGCTAAATCTACTGAACTA. The primers were analyzed using Net Primer (Premier Biosoft, San Francisco, CA, USA) to determine the melting temperature and secondary structures. The specificity of *S. frugiperda* primers was tested in two ways. First, sequences in GenBank from *Spodoptera dolichos* (access numbers: JQ559274.1, HQ568393.1, and KJ634288.1), *Spodoptera eridania* (access numbers: KJ634289.1, JQ551023.1, and KF261171.1), *Spodoptera cosmioides* (access numbers: JF854980.1 and KF261200.1), *Spodoptera albula* (access numbers: KF261151.1, KF261154.1, GU658150.1, and JF855904.1), and *E. kuehniella* (access number: GU828613.1) were aligned with the designed primers in software Sequencher 4.0.1 (Gene Codes Corp., Ann Arbor, MI, USA) (Figure 1). Second, the specificity of the primers was tested with PCR assays with DNA from *H. convergens*, *D. luteipes*, *H. axyridis*, *Cycloneda sanguinea*, *Anticarsia gemmatalis*, *Helicoverpa zea*, *Dalbulus maidis*, and *E. kuehniella* and four other *Spodoptera* species, *S. albula*, *S. cosmioides*, *S. dolichos*, and *S. eridania*.

The PCR assays for this study were optimized in a total volume of 14 µL, with 7.1 µL of DNA, 1.5 µL of 10× magnesium-free buffer (Sinapse Inc., Hollywood, FL, USA), 1.5 µL MgCl2 (25 mM, Sinapse Inc.), 1.2 µL dNTP (2.5 mM, Sinapse Inc.), 1.2 µL of each primer (5 pmol), and 0.3 Taq DNA polymerase 5U/µL (Sinapse Inc.). The conditions used in the PCR assay were: 94 °C for 3 min; then 35 cycles of 94 °C for 30 s, 57 °C for 45 s, 72 °C for 2 min; and 72 °C for 10 min for the final extension in the Veriti™ 96-Well Thermal Cycler (Applied Biosystems, Waltham, MA, USA). The amplification (fragment size: 130 bp) was evaluated by electrophoresis on 2% agarose gel. PCR assays to detect the presence of *S. frugiperda* DNA in predator gut contents were carried out simultaneously with three control samples: water and DNA predators that did not have contact with *S. frugiperda* as two negative controls, and a positive control DNA of *S. frugiperda*.

#### 2.2.3. Feeding Studies

Adults of *H. convergens* were sampled in a maize field in Pirassununga (22°03′59.8″ S 47°25′58.4″ W), São Paulo state, Brazil. The ladybugs were reared in plastic Petri dishes for oviposition and fed with inviable eggs of *Ephestia kuehniella* Zeller (Lepidoptera: Pyralidae) and a mixture of water, honey, and brewer’s yeast in cotton. *H. convergens* eggs were removed to new containers and held until the larvae emerged. The larvae were kept in glass tubes and fed with inviable eggs of *E. kuehniella* until they reached the fourth instar. All adults and larvae were reared in Bio-Oxygen Demand (BOD) incubators with a photoperiod regime of 14:10 (L:D) and 60 ± 10% relative humidity (RH) at 26 ± 1 °C.

*Doru luteipes* adults were collected in the field and maintained in dark plastic containers, with artificial diet that contained 35% cat food, 27% wheat germ, 23% brewer’s yeast, 14% milk in powder, 0.5% nipagin, and 0.5% sorbic acid [30]; and an egg box, folded paper, and plastic straws with moistened cotton on one side. The adults oviposited in the moist cotton, and the straws with the eggs and the mother earwigs were maintained in another container until the nymphs reached the second instar. All the containers were kept in a climate-controlled room with 70 ± 10% relative humidity (RH) at 26 ± 1 °C in the dark.

A total of 70 *H. convergens* fourth-instar larvae and 66 *D. luteipes* nymphs were starved for 24 h. They were placed in individual Petri dishes and were offered a single *S. frugiperda* neonate larva for each individual of *H. convergens* and *D. luteipes*. The predators were transferred to microtubes containing 99.9% ethanol after 0.5, 2, 4, 12, 24, and 48 h of feeding on *S. frugiperda* and stored at –20 °C. During the period between the feeding and transfer to microtubes, the individuals were kept with no food in a BOD incubator. Each time group of *H. convergens* contained 10 individuals. Groups 0–12 h of *D. luteipes* contained 10 individuals, the 24 h group contained 9, and the 48 h group contained 7. The DNA extraction and PCR to detect the presence or absence of *S. frugiperda* DNA in the predators’ gut contents were carried out according to the methods described above. Additionally, during these bioassays, some positive PCR fragments were sequenced by Sanger sequencing to confirm that the amplified fragment with these primers corresponded only to *S. frugiperda*.

The feeding results were analyzed by using the package “drc” in R Studio [31,32] to determine the half-life of the DNA in the predators’ gut contents.

## 3. Results

### 3.1. Primer Specificity and Half-Life for S. frugiperda DNA

**Figure 2:**
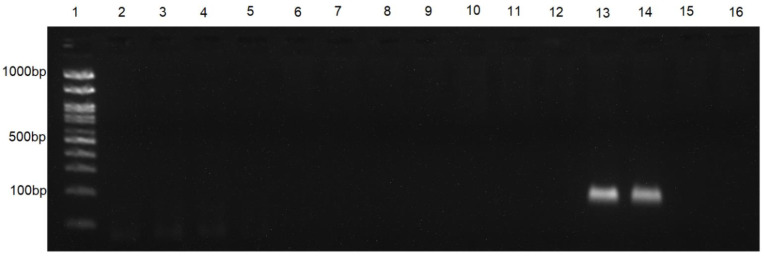
Electrophoresis gel showing the specificity of the primers Spo_frugi-F and Spo_frugi-R when tested with a PCR assay using DNA from different predators, months, and *Spodoptera frugiperda*. Column 1, ladder; columns 2–12, DNA of *Hippodamia convergens, Doru luteipes, Harmonia axyridis, Cycloneda sanguinea, Anticarsia gemmatalis, Helicoverpa zea, Dalbulus maidis, Ephestia kuehniella, Spodoptera albula, Spodoptera cosmioides,* and *Spodoptera eridania*, respectively. Column 13, DNA of *S. frugiperda*; column 14, gut contents of *H. convergens* that had fed on *S. frugiperda*; column 15, DNA of *Spodoptera dolichos*; column 16, negative control.

The primers Spo_frugi-F and Spo_frugi-R designed for *S. frugiperda* were highly species-specific in our PCR conditions, and they were able to only amplify the target species when tested with DNA from other species of *Spodoptera* and other moths, earwigs, and ladybugs (Figure 2). Only predators that had fed on *S. frugiperda* in the laboratory (positive control) had a positive amplification in the PCR assay. The half-life detection time (DT_50_) for *S. frugiperda* DNA in *H. convergens* gut contents was DT_50_ = 6.16 h (± 1.46, t = 4.21, *p* < 0.01), and in *D. luteipes*, it was DT_50_ = 25.72 h (±2.94, t = 8.73, *p* > 0.001).

### 3.2. Insect Field Collection and Predator Gut-Content Analysis

**Figure 3:**
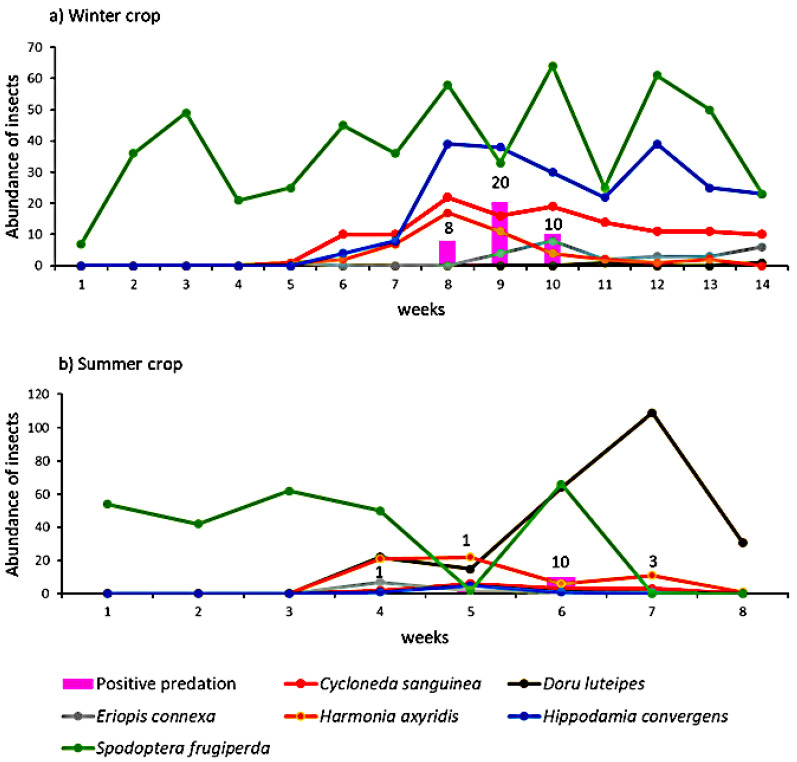
Total number of individuals of *Doru luteipes, Hippodamia convergens, Harmonia axyridis, Cycloneda sanguinea,* and *Eriopis connexa* sampled per week in Pirassununga during (**a**) winter crop (April to July 2020) and (**b**) summer crop (December 2020 to February 2021). Pink bars and the numbers on the top of the bars represent the total predation events identified as a positive result in PCRs from the field samples per week. The lines with dots represent the predator species sampled per week in the field.

In the winter crop, a total of 388 predators were sampled. Of these, *H. convergens* was the most abundant predator with 228 samples collected, followed by 124 for *C. sanguinea*, 47 for *H. axyridis*, 27 for *Eriopis connexa*, and 2 for *D. luteipes*. The predators appeared in week 5 with 3 ladybugs; week 6 with 16 ladybugs; week 7 with 25 ladybugs; week 8 with 78 ladybugs; week 9 with 69; week 10 with 61; week 11 with 40 ladybugs and 1 earwig; week 12 with 54 ladybugs; week 13 with 41 ladybugs; and week 14 with 39 ladybugs and 1 earwig (Figure 3a). The weeks that were most abundant in predators were weeks 8 and 9 when the maize was in the beginning of reproductive stage. The populations of *H. axyridis* and *C. sanguinea* fluctuated similarly, reaching their highest in week 8 and decreasing continuously in the following weeks. The population of *E. connexa* fluctuated constantly during the maize crop cycle (Figure 3a).

Predation events in the winter crop occurred only in weeks 8, 9, and 10, and all ladybug species fed on *S. frugiperda*. *H. convergens* had the most positive results (24 positives) with predation rates varying between 5 and 42%, followed by *H. axyridis* (6 positives) with predation rates between 11 and 36%. The predation rate for *C. sanguinea* (four positives) was 18.2% and for *E. connexa* (two positives) was 25% (Table 1).

In the summer crop, a total of 334 predators were sampled, of which 241 were *D. luteipes* and 93 were ladybugs. The most abundant ladybug species was *H. axyridis*, with 61 individuals (Figure 3b). The predators appeared in week 4, at the end of vegetative stage, with 31 ladybugs and 22 earwigs; week 5 with 35 ladybugs and 15 earwigs; week 6 with 11 ladybugs and 64 earwigs; week 7 with 15 ladybugs and 109 earwigs; and week 8 with 1 ladybug and 31 earwigs. The most abundant weeks were 6 and 7 for earwigs, and weeks 4 and 5. Predation events were detected in 14 *D. luteipes*, with the predation rate varying between 1% to 9%. No predation was detected in ladybug individuals. One predation event occurred in the maize vegetative stage, week 4, and three predation events in reproductive stage, weeks 5, 6, and 7, where week 6 had the most predation events (Table 1).

## 4. Discussion

We developed a molecular marker for the specific identification of *S. frugiperda* in predator gut contents. This marker can distinguish *S. frugiperda* from other *Spodoptera* species and is useful for identifying and monitoring this cosmopolitan pest by means of a single PCR method. The *S. frugiperda*-specific primers were based on the *cytochrome c oxidase subunit I* (COI) gene and were successfully used as a DNA barcoding region in animals [33,34,35,36]. One advantage of the molecular marker developed here is the small fragment size amplified, favoring PCR amplification and identification of insects even when the DNA was degraded, which is an essential trait of a molecular marker for gut-content analysis studies [28,37].

The DNA half-life for detection of *S. frugiperda* in *H. convergens* and *D. luteipes* gut contents differed between predators, with rates of 6.16 h and 25.72 h for *H. convergens* and *D. luteipes,* respectively. DNA half-life for detection of other prey in *H. convergens* gut contents showed a similar detection time for *Rhopalosiphum maidis* (Hemiptera: Aphydiidae), 8.78 h [28]; and for *Diaphorina citri* (Hemiptera: Psyllidae), 6.11 h [38]. Here, the DNA half-life detection time in gut contents was estimated for the first time in *D. luteipes*, and it was longer than in *H. convergens*.

Differences in DNA half-life detection times in predator gut contents are expected because the DNA half-life is driven by environmental conditions and by biological traits of the predator [39,40]. Insect life stage, metabolism, mouthpart type, and gut size and shape are some variables that affect the process of DNA degradation in the predator gut and, consequently, the DNA half-life detection time [27,38,41]. However, DNA half-life detection assays in laboratory conditions are useful and essential for determining if the molecular marker is efficient in detecting low concentrations of prey DNA in predator gut contents, as we confirmed here for this *S. frugiperda* marker.

Predation on *S. frugiperda* was detected in all predator species collected in maize fields using DNA gut-content analysis. However, the higher predation rates were detected in *H. convergens* and *H. axyridis*—even higher than in *D. luteipes,* reported as an efficient predator of *S. frugiperda* [16]. The higher predation rate detected in these ladybug species compared with *D. luteipes* is likely even higher, in view of the 4.0x longer DNA half-life in gut contents of *D. luteipes* compared to *H. convergens* in laboratory conditions. Therefore, ladybugs must be considered as biological-control agents for *S. frugiperda*, especially *H. convergens* and *H. axyridis*, the two most abundant coccinellid species in our samples.

In the field, generalist predators can feed on a variety of sources such as pollen, decomposing animal and plant tissues, and different prey species. High rates of prey consumption by a predator in the laboratory or greenhouse, as reported for *D. luteipes* on *S. frugiperda* [16], may not indicate a high predation rate in the field because it also commonly exhibits scavenging behavior. Alternative prey types or feeding behavior may also affect the predation rate of a predator on a target prey [42,43]. For example, ladybugs may prefer to feed on aphids rather than other prey, as these predators are commonly reported as biological-control agents for aphids [44].

The presence of *S. frugiperda* in the field was constant throughout the maize cycle. This species damages leaves and cobs, but maize is the most susceptible in the vegetative and initial reproductive stages, when *S. frugiperda* may feed on the meristem of young plants, stunting or killing them, and damage the kernels, causing enormous losses [45]. However, in both collection seasons, the predators were found in the area from six weeks after the maize sprouted, and their peak was in the maize reproductive stage. Predators are also attracted to the plant by the pollen, nectar, and synomones [46,47]. At the beginning of the reproductive stage, maize produces pollen that attracts insects, including different predator species [48,49,50]. This delay in the arrival of predators, observed in both seasons in comparison to *S. frugiperda*, may reduce the predators’ control efficiency when the maize is most susceptible to the pest. Thus, strategies to advance their arrival and retain the predators in the area should be adopted to improve the biological control of *S. frugiperda* in the first weeks of plant development [42,51,52].

## 5. Conclusions

We successfully constructed a molecular marker to detect *S. frugiperda* DNA in predator gut contents. Using this molecular marker, we confirmed that earwigs and ladybugs, especially *H. convergens* and *H. axyridis*, preyed on *S. frugiperda* in the maize field. Our study indicated that predators might be considered a reliable component of IPM for *S. frugiperda*. Therefore, a better understanding of the dynamics of prey and predator in the landscape is crucial for managing predators and implementing conservative and applied biological control programs in maize fields.

## Figures and Tables

**Table 1:** Weekly abundance and predation rates for each predator that showed positive results for predation on *Spodoptera frugiperda* in winter and summer crops.

	Winter Crop						Summer Crop							
	Week 8		Week 9		Week 10		Week 4		Week 5		Week 6		Week 7	
Predator	Ab ^1^	P.R ^2^	Ab	P.R	Ab	P.R	Ab	P.R	Ab	P.R	Ab	P.R	Ab	P.R
*Hippodamia convergens*	39	5.1± 0.06	38	42.1 ± 0.156	30	26.7± 0.158	1	-	5	-	1	-	-	-
*Harmonia axyridis*	17	11.8 ± 0.153	11	36.4± 0.284	4	-	21	-	22	-	6	-	11	-
*Cycloneda sanguinea*	22	18.2± 0.161	16	-	19	-	2	-	6	-	3	-	3	-
*Eriopis connexa*	-	-	4	-	8	25± 0.3	7	-	1	-	1	-	1	-
*Doru luteipes*	-	-	-	-	-	-	22	4.5± 0.087	15	6.7± 0.126	64	1.6± 0.088	109	9.2± 0.03

^1^ Ab = abundance; ^2^ P.R (%) = predation rates in percentage ± IC (confidence interval).

## Data Availability

All relevant data are within the paper.
